# London University and the Royal Colleges

**Published:** 1910-04-02

**Authors:** 


### London University and the Royal Colleges.

Whatever may be the upshot of the agitation which has been steadily gathering force in recent years for the acquisition of London degrees by those whose standard of attainments, as measured by examinations, has hitherto procured for them only the licence of the two Royal Colleges, it cannot be said that either party to the negotiations—the University, that is to say, and the Corporations—is now dilatory or unreasonable. At the instance of the Senate of London University a Royal Commission has been granted to consider the matter, and the delegates originally requested by the Colleges to draw up a draft scheme for submission to the Senate have in consequence quite properly taken this Commission into account, and have presented to their respective elective bodies a report setting forth what evidence should, in their opinion, be offered before the Commission on behalf of the Joint Examining Board. In this report are sketched out several heads under which it is sought to establish by statistics the disability from which the London medical student labours by not being able to obtain a degree of M.D. on such terms as exist in other universities in the United Kingdom. So far as this outline has yet been drawn, there seems to be nothing very novel about it, nor, indeed, very convincing either. It fails to take into account two of the most vital factors in the problem, on which insistence has on a previous occasion been laid in these columns. One of these is that the London degrees, especially the pass degrees, are not in the least beyond the capacity, mental or financial, of the average student who will consent to work for them; and, sequentially, the other is that it is because these degrees are denied to the least able and least hardworking that their present high reputation has rendered them so valuable. The draft scheme endeavours to surmount this difficulty by providing separate curricula, courses, and examinations (even separate examining boards too) for the pass and honours degrees respectively. But this way out ignores the patent fact that the public, which discriminates easily enough between M.D. and M.R.C.S., cannot naturally grasp so readily the distinctions between M.D. (pass) and M.D. (honours). Moreover, in practice it would inevitably come about that all London M.D.s—whether pass or honours men—would be described simply as M.D. without the cumbrous and rather invidious differentiation conferred by the qualifying adjective. Whether this is the contemplated and desired object of the delegates of the Royal Colleges we do not know. It is at any rate not their avowed intention; but it is very clear what the result would be. The honours course would be of no extra value to the general practitioner, since it would give him no extra *cachet* in the estimation of his patients. It might become semi-compulsory for the budding consultant ambitious to be elected to the staff of a London hospital, and, therefore, it would not be altogether abandoned. But it seems fairly certain that as far as ordinary general practice is concerned the honours degrees would lapse altogether and be quite neglected. In addition to that, the degrees as a whole, honours and pass alike, would suffer a great fall in public estimation, to the detriment of all the present holders of them. Medical authorities in London appear to realise very imperfectly the extraordinary prestige which the London M.D. carries with it among the public at large. That this is greater, perhaps, than in the strictest justice it always deserves may be true; but it does not alter the fact of its existence, and to level down degrees in London to the standard of the mushroom universities of the provinces is to drop a very substantial and tangible asset for an extremely shadowy and hypothetical advantage. It is doubtful whether this is the best means of restoring financial prosperity to the London schools and licensing bodies. The clinical opportunities of London are unrivalled; as long as the teaching remains the same London's popularity as a medical centre must soon return.

